# Targeting Cytokine Dysregulation in Psoriasis: The Role of Dietary Interventions in Modulating the Immune Response

**DOI:** 10.3390/ijms26072895

**Published:** 2025-03-22

**Authors:** Daniel Simancas-Racines, Náthaly Mercedes Román-Galeano, Ludovica Verde, Giuseppe Annunziata, Marco Marchetti, Andri Matos, Martín Campuzano-Donoso, Claudia Reytor-González, Giovanna Muscogiuri, Luigi Barrea, Evelyn Frias-Toral

**Affiliations:** 1 Universidad UTE, Facultad de Ciencias de la Salud Eugenio Espejo, Centro de Investigación en Salud Pública y Epidemiología Clínica (CISPEC), Quito 170527, Ecuador; dsimancas@ute.edu.ec (D.S.-R.); nathalyroman0001@gmail.com (N.M.R.-G.); martincd01@hotmail.com (M.C.-D.); 2 Department of Public Health, University of Naples Federico II, Via Sergio Pansini 5, 80131 Naples, Italy; ludovica.verde@unina.it; 3 Facoltà di Scienze Umane, della Formazione e dello Sport, Università Telematica Pegaso, Via Porzio, Centro Direzionale, Isola F2, 80143 Naples, Italy; giuseppe.annunziata@unipegaso.it; 4 Departmental Faculty of Medicine, UniCamillus-Saint Camillus International University of Health Sciences, Via Di Sant’Alessandro 8, 00131 Rome, Italy; marco.marchetti@unicamillus.org; 5 School of Allied Health, Eastwick College, Ramsey, NJ 07446, USA; amatos@eastwick.edu; 6 Unit of Endocrinology, Dipartimento di Medicina Clinica e Chirurgia, Federico II University Medical School of Naples, Via Sergio Pansini 5, 80131 Naples, Italy; giovanna.muscogiuri@unina.it; 7 Cattedra Unesco “Educazione Alla Salute e Allo Sviluppo Sostenibile”, University Federico II, Corso Umberto I 40, 80131 Naples, Italy; 8 Dipartimento Psicologia e Scienze della Salute, Università Telematica Pegaso, Centro Direzionale Isola F2, Via Porzio, 80143 Naples, Italy; luigi.barrea@unipegaso.it; 9 Escuela de Medicina, Universidad Espíritu Santo, Samborondón 0901952, Ecuador; 10 Division of Research, Texas State University, 601 University Dr, San Marcos, TX 78666, USA

**Keywords:** psoriasis, cytokines, obesity, dietary interventions, Mediterranean diet, ketogenic diet, fasting-mimicking diet, inflammation, personalized nutrition

## Abstract

Psoriasis is a chronic immune-mediated skin disease characterized by cytokine dysregulation. Pro-inflammatory mediators, including tumor necrosis factor-alpha (TNF-α), interleukin (IL)-17, and IL-23, play pivotal roles in the pathogenesis of psoriasis. Emerging evidence suggests that dietary interventions can modulate cytokine activity, providing a complementary approach to standard therapies. This narrative review examines the impact of various dietary strategies, including a Mediterranean diet, ketogenic diet, gluten-free diet, and fasting-mimicking diet, on cytokine profiles and clinical outcomes in psoriasis. Research insights reveal that dietary components such as omega-3 fatty acids, polyphenols, and short-chain fatty acids influence immune signaling pathways. These pathways include nuclear factor-kappa B (NF-κB) and Signal Transducer and Activator of Transcription 3 (STAT3). Additionally, these dietary components promote anti-inflammatory effects mediated by gut microbiota. Clinical studies demonstrate significant reductions in psoriasis severity, improved quality of life, and modulation of key cytokines associated with disease activity. Despite these advancements, significant challenges persist in effectively integrating these findings into clinical practice. These challenges include variability in patient responses, adherence issues, and the need for robust biomarkers to monitor efficacy. Future directions emphasize the potential of personalized nutrition and precision medicine approaches to optimize dietary interventions tailored to individual cytokine profiles and genetic predispositions. Integrating these strategies into psoriasis care could transform treatment paradigms by simultaneously addressing both systemic inflammation and comorbid conditions.

## 1. Introduction

Psoriasis is a chronic immune-mediated skin disorder that manifests as well-demarcated erythematous plaques covered with silvery scales. These plaques result from hyperproliferation and abnormal differentiation of keratinocytes, accompanied by a dysregulated immune response [1]. Beyond its cutaneous manifestations, psoriasis is increasingly recognized as a systemic inflammatory condition, with a heightened risk of comorbidities such as cardiovascular diseases, metabolic syndrome, type 2 diabetes, and depression. Additionally, psoriasis significantly impacts patients’ quality of life, as demonstrated by studies from Scala et al. and Balato et al., which emphasize the influence of sociodemographic, clinical, and therapeutic factors [2,3]. These associations underscore the importance of addressing not only the skin manifestations but also the underlying inflammatory mechanisms driving the disease [4,5,6,7].

Cytokine dysregulation is central to the pathogenesis of psoriasis, with a network of pro-inflammatory and anti-inflammatory mediators shaping its onset, progression, and severity [8]. Among the most prominent cytokines involved are tumor necrosis factor-alpha (TNF-α), interleukin (IL)-17, IL-23, and IL-22, which play critical roles in the inflammatory cascade [1,4].

These cytokines orchestrate the recruitment and activation of immune cells, notably T-helper 17 (Th17) cells and dendritic cells, creating a self-perpetuating inflammatory loop [9]. Consequently, biologic therapies targeting these cytokines have transformed psoriasis management, delivering significant clinical improvements [10]. However, these therapies come with limitations, including high costs, risk of adverse effects, and variability in patient response, underscoring the need for complementary strategies [11,12,13]. In recent years, dietary interventions have garnered attention for their potential to modulate immune responses and inflammatory pathways [7,14,15,16], providing a cost-effective and holistic approach to managing chronic diseases such as psoriasis [17]. Emerging evidence suggests that dietary patterns and specific nutrients can directly influence cytokine production and immune function [18,19,20,21]. For example, the Mediterranean diet, which is rich in omega-3 fatty acids and polyphenols, has demonstrated anti-inflammatory effects that could potentially reduce the activity of key cytokines such as IL-17 and TNF-α [22]. Similarly, ketogenic diets, gluten-free diets, and caloric restriction show considerable promise in modulating immune pathways and improving psoriasis outcomes [23,24,25]. These dietary strategies align with a growing emphasis on lifestyle interventions as adjunctive tools in chronic disease management [26,27,28].

Despite the increasing interest in diet–immune interactions, the mechanisms by which dietary interventions influence cytokine dysregulation in psoriasis are not yet fully elucidated. Factors such as nutrient-specific effects on signaling pathways, the role of gut microbiota, and interindividual variability pose challenges to the translation of research findings into clinical practice. Additionally, while some studies highlight the benefits of dietary interventions, others report inconsistent or limited effects, emphasizing the need for further exploration.

This narrative review aims to provide a comprehensive examination of the role of dietary interventions in modulating cytokine dysregulation in psoriasis. It seeks to elucidate the molecular mechanisms through which diet influences immune function, summarize current evidence linking specific dietary patterns to changes in cytokine profiles, and discuss the clinical implications of these findings. By integrating insights from preclinical and clinical studies, this review also highlights challenges in applying dietary strategies in practice and identifies future research directions, such as personalized nutrition and precision medicine approaches, to optimize psoriasis management. Ultimately, this review highlights the potential of dietary interventions as a valuable component of a multidisciplinary approach to managing psoriasis and its associated systemic inflammation.

## 2. Cytokine Dysregulation in Psoriasis

**Figure 1:**
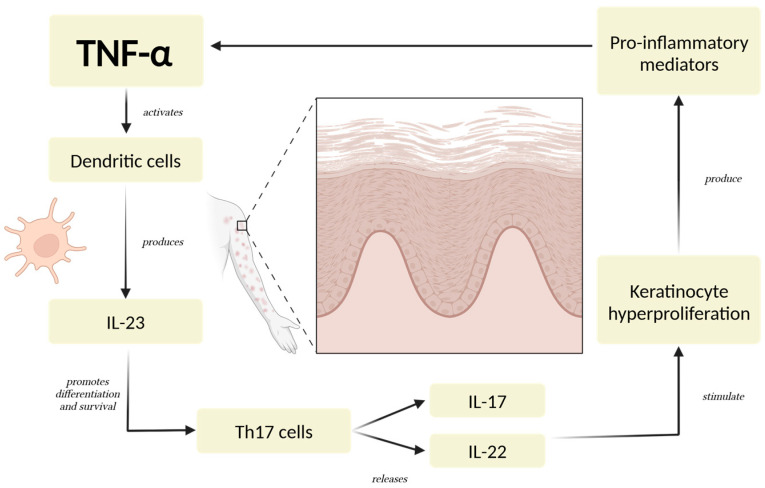
The Psoriatic Inflammatory Loop. TNF-α triggers a series of immune responses involving dendritic cells, Th17 cells, and keratinocytes. These cells, through the production of critical cytokines like IL-23, IL-17, and IL-22, establish a persistent pro-inflammatory state [4,30]. The cycle is reinforced as keratinocytes further stimulate immune cell activation and recruitment, intensifying the inflammatory signaling. This mechanism leads to excessive keratinocyte proliferation, a defining feature of psoriatic lesions, and sustains the chronic nature of the condition [10,32]. Abbreviations: TNF-α: tumor necrosis factor-alpha; IL-23: interleukin-23; IL-17: interleukin-17; IL-22: interleukin 22.

Dysregulated cytokine activity plays a key role in the development and progression of psoriasis [29]. Among the main cytokines involved, TNF-α serves as a pivotal pro-inflammatory mediator, initiating and regulating a cascade of immune responses [4,30]. TNF-α contributes to the activation of dendritic cells, which subsequently produce IL-23, a cytokine that sustains the differentiation and survival of Th17 cells [31]. These Th17 cells secrete IL-17 and IL-22, driving keratinocyte hyperproliferation and amplifying the inflammatory loop characteristic of psoriatic lesions [10,32,33] (Figure 1).

IL-17 is one of the most important cytokines in psoriasis, as it directly promotes inflammation and keratinocyte activation [34,35]. It enhances the expression of antimicrobial peptides, pro-inflammatory cytokines, and chemokines, contributing to neutrophil recruitment and the creation of a psoriatic plaque [36,37]. IL-23 is equally important, as it maintains the Th17 cell population and supports IL-17 production, making it a regulator in the disease process [38,39,40]. IL-22, another cytokine associated with the Th17 axis, plays a role in epidermal hyperplasia and barrier dysfunction, further exacerbating the clinical manifestations of psoriasis [41,42].

Pro-inflammatory cytokines like TNF-α, IL-17, and IL-23 are carefully regulated in healthy individuals by anti-inflammatory cytokines and regulatory mechanisms [43]. However, in psoriasis, this balance is disrupted, resulting in unchecked inflammation [30,36]. Regulatory cytokines such as IL-10 and transforming growth factor-beta (TGF-β) are often suppressed or functionally impaired in psoriatic patients [44]. This suppression reduces the immune system’s ability to resolve inflammation, contributing to chronic disease progression and comorbidities [38].

The clinical manifestations of psoriasis, including erythematous plaques, scaling, and systemic inflammation, are closely linked to cytokine activity [45]. For example, elevated levels of TNF-α and IL-17 correlate with disease severity as measured by the Psoriasis Area and Severity Index (PASI) [46,47]. The inflammatory microenvironment created by cytokines affects the skin and systemically propagates inflammation, increasing the risk of conditions such as cardiovascular disease, metabolic syndrome, and psoriatic arthritis [4,10]. This systemic involvement highlights the far-reaching impact of cytokine dysregulation beyond visible skin lesions.

Advances in cytokine research have transformed the treatment of psoriasis, paving the way for targeted biologic therapies. Biologics that inhibit TNF-α, IL-17, or IL-23 have shown significant efficacy in reducing inflammation and clearing psoriatic lesions [30,32,40]. However, disparities in access to biologic therapies have been observed, with socioeconomic, geographic, and demographic factors influencing treatment decisions [48]. A study by Scala et al. highlighted that patients from lower socioeconomic backgrounds, as well as older individuals and those with obesity, were less likely to receive biologic treatments, despite their proven efficacy in controlling cytokine-driven inflammation [49]. These findings underscore the need for a more equitable approach to psoriasis management, considering not only pharmacological interventions but also complementary strategies such as dietary modifications to modulate cytokine activity and achieve holistic disease control.

## 3. Dietary Therapies in Psoriasis: An Overview

Dietary interventions are increasingly recognized as complementary strategies to manage psoriasis, given the disease’s strong links to systemic inflammation and metabolic dysregulation [7,50,51,52]. Various dietary patterns have been studied for their potential to modulate immune responses and inflammatory pathways, influencing psoriasis severity and progression [28,53,54].

Among these, the Mediterranean diet stands out for its anti-inflammatory properties [55,56,57]. This diet prioritizes whole grains, fruits, nuts, vegetables, seeds, and olive oil, while moderating fish intake and minimizing red meat and processed foods [58]. Observational studies and clinical trials consistently demonstrate that adherence to anti-inflammatory diets, such as the Mediterranean diet, significantly reduces disease severity and enhances quality of life [59,60,61,62]. Plant-based diets, rich in phytonutrients, antioxidants, and dietary fibers, provide anti-inflammatory benefits that modulate cytokine activity in psoriasis. These diets reduce oxidative stress, promote regulatory T-cell function, and decrease pro-inflammatory cytokines such as TNF-α and IL-17 [50]. Additionally, specific dietary exclusions, such as gluten-free or low fermentable oligosaccharides, disaccharides, monosaccharides and polyols diets (low-FODMAP diets), have demonstrated potential benefits in specific patients with autoimmune-related diseases [63].

Another dietary strategy that has gained attention is the ketogenic diet, characterized by high fat and low carbohydrate intake. Small studies suggest that this diet may reduce psoriatic lesions by lowering systemic inflammation and modifying metabolic pathways [27]. By inducing ketosis, the ketogenic diet modulates the activity of regulatory T cells and decreases the expression of inflammatory cytokines, contributing to an overall anti-inflammatory effect [64,65,66].

The mechanisms by which diet influences psoriasis involve multiple pathways, particularly those related to immune regulation and cytokine production [67]. Diets rich in omega-3 fatty acids, such as the Mediterranean diet, can inhibit the production of pro-inflammatory cytokines like TNF-α and IL-17. These diets promote anti-inflammatory mediators [68]. Polyphenols and antioxidants found in plant-based foods modulate oxidative stress and improve immune cell function [58,69]. Additionally, modulation of gut microbiota plays a crucial role in linking diet to psoriasis. Dietary components such as fiber, polyphenols, and fermented foods shape the gut microbiome, which is essential for immune homeostasis. Healthy microbiota produces metabolites like short-chain fatty acids (SCFAs) that enhance regulatory T cell activity and reduce inflammation [16,70]. Since dysbiosis is often observed in psoriasis, targeted dietary interventions may provide a strategy to improve disease symptoms and progression [64,65,66,71,72].

Despite promising findings, translating dietary therapies into standardized recommendations for psoriasis management remains challenging [16,70,71,72]. Interindividual variability, influenced by genetics, comorbidities, and adherence, underscores the need for personalized nutrition approaches [73,74,75,76]. Future research should focus on large-scale clinical trials to determine the efficacy of specific dietary patterns and identify response biomarkers [77]. Integrating dietary strategies with pharmacological and lifestyle interventions may allow clinicians to offer holistic care that addresses both the cutaneous and systemic dimensions of psoriasis [73,74,75,76,78,79].

## 4. Impact of Dietary Interventions on Key Cytokines in Psoriasis

### 4.1. Mediterranean Diet

The Mediterranean diet, characterized by its rich intake of fruits, vegetables, whole grains, olive oil, and moderate fish consumption, has demonstrated significant anti-inflammatory effects relevant to psoriasis [80,81,82]. This dietary pattern effectively modulates key cytokines involved in psoriasis pathogenesis, including IL-17, IL-23, and TNF-α [58]. By reducing the levels of these pro-inflammatory cytokines, the Mediterranean diet attenuates the chronic inflammatory state that underpins the disease [83]. Studies have shown that adherence to this diet correlates with lower psoriasis severity and a decreased systemic inflammatory burden, as measured by markers like C-reactive protein (CRP) and cytokine profiles [51].

Omega-3 fatty acids, abundant in fatty fish and nuts, play a crucial role in the anti-inflammatory effects of the Mediterranean diet [84]. These essential fatty acids inhibit the production of pro-inflammatory cytokines like TNF-α and IL-17 while promoting the synthesis of anti-inflammatory mediators such as resolvins and protectins [85]. Polyphenols, another key component found in olive oil, fruits, and vegetables, also contribute to cytokine modulation [86]. These bioactive compounds regulate the signaling pathways involved in immune responses, reducing the activation of dendritic cells and Th17 cells that drive IL-17 and IL-23 production. Together, these nutrients create a synergistic effect that directly impacts the immune dysregulation in psoriasis [87].

Clinical studies further support the link between the Mediterranean diet and improved cytokine profiles in psoriasis. For instance, patients who adopt this diet frequently report reductions in psoriatic lesion severity and improvement in systemic inflammation markers [88]. These benefits likely result from the diet’s ability to restore the balance between pro-inflammatory and anti-inflammatory cytokines, thereby achieving immune homeostasis. Additionally, the Mediterranean diet’s emphasis on whole unprocessed foods helps mitigate metabolic comorbidities like obesity, which exacerbate cytokine dysregulation [89,90].

### 4.2. Ketogenic Diet

The ketogenic diet, characterized by high-fat and low-carbohydrate intake, has demonstrated potential to modulate immune responses and inflammation, making it a promising dietary intervention for psoriasis [27,91]. By inducing a state of ketosis, where ketone bodies such as beta-hydroxybutyrate serve as an alternative energy source, the ketogenic diet exerts significant anti-inflammatory effects. These effects include the downregulation of pro-inflammatory cytokines like TNF-α, IL-17, and IL-1β, which are central to psoriasis pathogenesis [1]. Ketone bodies act as signaling molecules that inhibit NLRP3 inflammasome activation and reduce oxidative stress, thereby influencing the inflammatory pathways driving the disease.

Clinical outcomes in psoriasis patients following a ketogenic diet have been encouraging. Studies report reductions in the severity of psoriatic lesions, improved Psoriasis Area Severity Index (PASI) scores, and decreased systemic inflammation markers [92]. The diet’s high-fat composition, particularly from sources like omega-3-rich fish and monounsaturated fats, contributes to its efficacy by modulating regulatory immune pathways. For instance, the ketogenic diet enhances the function of regulatory T cells (Tregs), which help restore immune balance and suppress the activity of pro-inflammatory Th17 cells [93]. This shift in immune regulation is believed to mitigate the chronic inflammatory state seen in psoriasis.

On a molecular level, the ketogenic diet’s influence extends to metabolic pathways that intersect with immune function [89]. Ketosis alters gene expression profiles associated with inflammation, enhances antioxidant responses, and reduces cytokine production. Additionally, the diet improves insulin sensitivity and reduces adipose tissue inflammation, which are known contributors to systemic cytokine dysregulation in psoriatic patients. While more robust large-scale studies are needed to validate these findings, the ketogenic diet presents a compelling adjunctive therapy for psoriasis, offering both direct anti-inflammatory effects and improvements in metabolic health.

### 4.3. Gluten-Free Diet and FODMAPs

A gluten-free diet is also being explored as a potential dietary intervention for psoriasis, particularly in patients with comorbid gluten sensitivity or celiac disease [63,94]. Gluten can trigger systemic inflammation in susceptible individuals by increasing intestinal permeability and promoting immune activation. This heightened immune response is often accompanied by elevated levels of pro-inflammatory cytokines, such as IL-6, TNF-α, and IL-17, which are also key players in psoriasis pathogenesis. By eliminating gluten-containing foods like wheat, barley, and rye, a gluten-free diet may help reduce systemic cytokine dysregulation and mitigate psoriasis severity [63].

Low-FODMAP diets, which restrict fermentable oligosaccharides, disaccharides, monosaccharides, and polyols, also show promise in cytokine modulation for psoriatic patients with gastrointestinal comorbidities such as irritable bowel syndrome (IBS) [95]. Dysbiosis, often exacerbated by FODMAP-rich foods, can lead to increased production of pro-inflammatory cytokines, including IL-6 and IL-1β [96]. By reducing gut inflammation and restoring microbiome balance, a low-FODMAP diet may indirectly influence systemic immune responses [95]. Clinical studies indicate that these dietary modifications are associated with reduced inflammatory markers and improvements in both skin symptoms and gastrointestinal health.

Although evidence of the efficacy of gluten-free diet and low-FODMAP diets in psoriasis is still emerging, initial studies suggest a beneficial role in cytokine modulation and symptom reduction. Both diets target the gut–skin axis, emphasizing the importance of gastrointestinal health in systemic inflammation and immune regulation [89]. For patients with overlapping gastrointestinal issues or heightened sensitivity to specific foods, these dietary approaches can provide a personalized strategy to reduce pro-inflammatory cytokines like IL-6 and TNF-α, offering relief from psoriatic symptoms and improving overall quality of life [97,98,99]. These findings are summarized in Table 1, which provides a comparative overview of dietary interventions, their mechanisms, and their impact on psoriasis pathogenesis.

### 4.4. Caloric Restriction and Fasting-Mimicking Diets

Caloric restriction (CR) and fasting-mimicking diets (FMDs) have gained attention for their significant impact on systemic inflammation and immune regulation, making them promising interventions for psoriasis management and other chronic conditions such as diabetes, neurodegenerative diseases, autoimmune diseases, and cancer [89,100]. These dietary approaches involve reducing caloric intake either continuously or intermittently to mimic the physiological effects of fasting without complete food deprivation [101]. Both strategies have been shown to modulate key cytokines involved in psoriasis, such as TNF-α and IL-1β, which are central to the inflammatory processes driving the disease [102]. By limiting caloric intake, these diets reduce the activation of pro-inflammatory pathways, including the NF-κB and NLRP3 inflammasome, thereby attenuating the production of these cytokines [103,104].

In addition to direct effects on cytokine production, CR and FMDs confer benefits by influencing metabolic pathways that contribute to chronic inflammation [101]. These diets enhance insulin sensitivity, reduce adiposity, and decrease levels of leptin, a pro-inflammatory adipokine that exacerbates immune dysregulation [105]. At the same time, fasting states stimulate the production of anti-inflammatory molecules such as adiponectin and ketone bodies, which help regulate immune responses and oxidative stress [106]. This metabolic shift is particularly relevant for psoriatic patients, as obesity and metabolic syndrome are common comorbidities that amplify cytokine dysregulation and disease severity [107].

Preliminary studies and clinical trials highlight the efficacy of CR and FMDs in improving psoriasis outcomes. Participants often report reductions in lesion severity, systemic inflammation markers, and comorbid metabolic issues [102]. These improvements are thought to result from the interplay between reduced pro-inflammatory cytokines and enhanced metabolic health. However, the sustainability and safety of these dietary interventions require further investigation, particularly in vulnerable populations. As research advances, CR and FMDs could become integral components of a holistic approach to managing psoriasis, targeting both immune and metabolic factors that drive disease progression [89]. These findings are summarized in Table 1, which provides a comparative overview of dietary interventions, their mechanisms, and their impact on psoriasis pathogenesis.

**Table 1:** Comparison of dietary interventions and their impact on psoriasis pathogenesis.

Dietary Intervention	Key Cytokines Affected	Mechanisms of Action	Clinical Outcomes	Additional Benefits
Mediterranean Diet	IL-17, IL-23, TNF-α	Modulates dendritic cell and Th17 activity; polyphenols and omega-3s reduce cytokine production and oxidative stress [87]	Reduces psoriasis severity, systemic inflammation, and cytokine levels [51]	Improves metabolic health and reduces obesity-related inflammation [89]
Ketogenic Diet	TNF-α, IL-17, IL-1β	Ketosis inhibits NLRP3 inflammasome; enhances regulatory T cells; alters inflammatory gene expression [93]	Improves PASI scores and decreases systemic inflammation [92]	Enhances insulin sensitivity, reduces oxidative stress and adiposity [91]
Gluten-Free Diet	IL-6, TNF-α, IL-17	Reduces intestinal permeability and immune activation in gluten-sensitive individuals [63]	Alleviates symptoms in patients with gluten sensitivity or celiac disease [40]	Targets gut–skin axis; improves gastrointestinal health [63]
Low-FODMAP Diet	IL-6, IL-1β	Restores microbiome balance; reduces gut dysbiosis and local inflammation [95]	Reduces gut-related symptoms and systemic inflammatory markers [89]	Benefits patients with IBS or other gastrointestinal comorbidities [95]
Caloric Restriction/FMDs	TNF-α, IL-1β	Decreases NF-κB and NLRP3 activity; enhances production of anti-inflammatory adiponectin and ketone bodies [103,104]	Reduces lesion severity, systemic inflammation, and metabolic comorbidities [107]	Promotes weight loss and insulin sensitivity [105]

Abbreviations: IL-17: Interleukin-17, IL-23: Interleukin-23, TNF-α: Tumor Necrosis Factor-alpha, IL-1β: Interleukin-1 beta, IL-6: Interleukin-6, NF-κB: Nuclear Factor kappa B, NLRP3: NOD-, LRR-, and pyrin domain-containing protein 3, FMDs: Fasting-Mimicking Diets, PASI: Psoriasis Area and Severity Index, IBS: Irritable Bowel Syndrome, Low-FODMAP Diet: Low fermentable oligosaccharides, disaccharides, monosaccharides and polyols diets.

## 5. Molecular Mechanisms Underpinning Cytokine Modulation by Diet

Dietary components exert relevant effects on cytokine signaling pathways, influencing both the onset and progression of inflammatory diseases like psoriasis [89]. Nutrients interact with cellular receptors, transcription factors, and enzymatic pathways to regulate the balance between pro-inflammatory and anti-inflammatory cytokines [108,109]. For instance, omega-3 fatty acids from fish oil inhibit the NF-κB signaling pathway, a key driver of pro-inflammatory cytokine production, while activating PPAR-γ, which promotes anti-inflammatory gene expression [110,111]. Similarly, polyphenols in fruits and vegetables modulate MAPK and JAK/STAT pathways, reducing the secretion of cytokines like TNF-α, IL-6, and IL-17 [112,113]. These mechanisms underline how diet can directly influence immune responses at the molecular level.

Nutrient-specific interactions play a critical role in shaping cytokine profiles. Omega-3 fatty acids are converted into specialized pro-resolving mediators (SPMs) such as resolvins and protectins, which actively suppress inflammation and downregulate IL-17 and IL-23 levels [114]. Conversely, high consumption of saturated fats and trans fats can stimulate Toll-like receptor signaling, leading to increased production of pro-inflammatory cytokines like IL-1β and TNF-α [115]. Dietary fiber, through its fermentation by gut bacteria, produces SCFAs such as butyrate, which enhance IL-10 production, an anti-inflammatory cytokine, while inhibiting pro-inflammatory cytokines [116]. These nutrient-cytokine interactions highlight the dual potential of diet to either exacerbate or mitigate inflammatory conditions.

A critical factor linking diet and immune function is gut microbiota composition. The Mediterranean diet and other fiber-rich dietary patterns promote the growth of beneficial bacteria such as *Faecalibacterium prausnitzii* and *Bifidobacterium*, which produce SCFAs that inhibit the production of pro-inflammatory cytokines like IL-6 and TNF-α, while promoting IL-10 [72,116]. Dysbiosis, often caused by high-fat or low-fiber diets, disrupts gut barrier integrity, allowing bacterial endotoxins, such as lipopolysaccharides, to enter the bloodstream and trigger systemic immune activation [96]. This results in increased cytokine production, contributing to inflammation in psoriasis.

Polyphenols and other bioactive compounds also interact with the gut microbiota to regulate cytokine production [117]. Polyphenols from foods like berries, tea, and olive oil serve as prebiotics, enhancing the growth of beneficial microbes that produce anti-inflammatory metabolites [113,118,119,120]. These metabolites, in turn, modulate cytokine signaling pathways, reducing the production of pro-inflammatory mediators. Additionally, polyphenols influence the differentiation of immune cells, such as Tregs and Th17 cells, which directly impact cytokine profiles [112]. This bidirectional interaction between dietary components and the microbiota highlights the importance of diet in maintaining immune homeostasis. Beyond polyphenols, other dietary components, such as fermented foods rich in probiotics, also play a key role in shaping gut microbiota composition and immune responses.

Fermented foods, including yogurt, kefir, sauerkraut, kimchi, and miso, play a crucial role in modulating the gut microbiota and immune response. These foods contain probiotics, which are live beneficial bacteria that colonize the gut and contribute to immune homeostasis [121]. Probiotics, particularly strains of *Lactobacillus* and *Bifidobacterium*, have been shown to downregulate pro-inflammatory cytokines such as TNF-α and IL-17 while enhancing the production of regulatory T cells (Tregs) [122]. Additionally, prebiotics, which are non-digestible fibers found in foods like garlic, onions, and bananas, promote the growth of beneficial gut bacteria, enhancing SCFA production and reducing systemic inflammation [123].

The combination of probiotics and prebiotics, known as synbiotics, has been explored as a potential dietary intervention for psoriasis. Synbiotics not only improve gut microbial balance but also enhance the survival and activity of probiotic strains, further amplifying their beneficial effects on immune modulation. By restoring gut microbiota diversity and function, these interventions help mitigate the chronic inflammatory state characteristic of psoriasis [124].

The interaction between diet and the gut–skin axis further supports the role of dietary interventions in psoriasis management [125]. The gut microbiome communicates with the immune system through microbial metabolites, which can either exacerbate or alleviate systemic inflammation. Studies suggest that gut dysbiosis in psoriatic patients is associated with altered microbial composition and reduced production of SCFAs, leading to an increased inflammatory response [126,127]. By integrating dietary strategies such as fermented foods, probiotics, prebiotics, and synbiotics, it is possible to target both gut and skin inflammation, providing a holistic approach to disease management.

**Figure 2:**
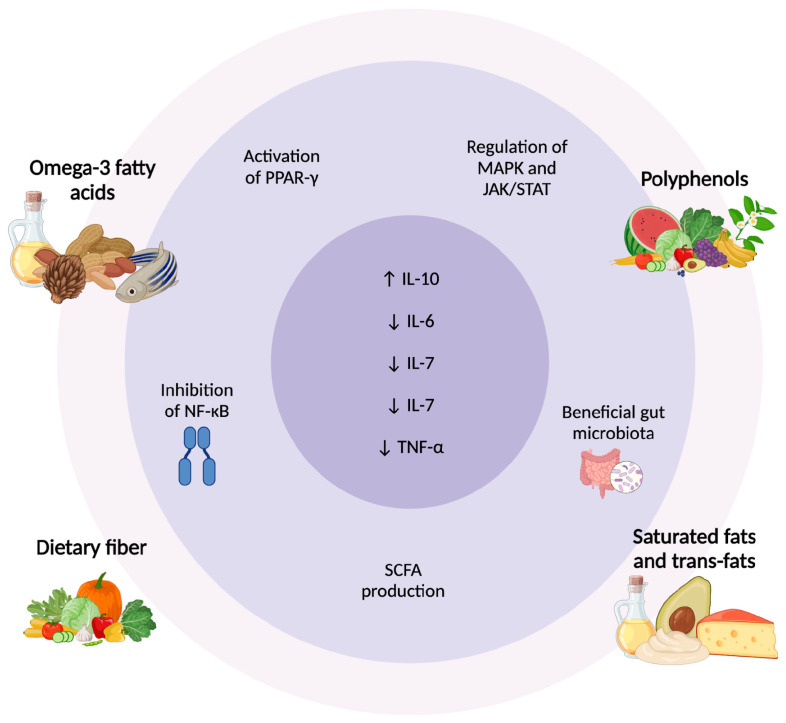
Dietary Regulation of Cytokines in Psoriasis: Molecular and Microbiota Pathways. Dietary components modulate inflammation in psoriasis through different pathways. Omega-3 fatty acids and polyphenols activate PPAR-γ, inhibit NF-κB, and regulate MAPK/JAK/STAT, reducing pro-inflammatory cytokines. Dietary fiber promotes SCFA production, enhancing anti-inflammatory responses. In contrast, saturated and trans fats contribute to gut dysbiosis and increased TNF-α, IL-6, and IL-7. These dietary influences highlight the role of nutrition in cytokine regulation and psoriasis management [108,109,112,116]. Abbreviations: ↑ Activation/increase; ↓ Inhibition/reduction; PPAR-γ, peroxisome proliferator-activated receptor gamma; NF-κB, nuclear factor kappa B; SCFA, short-chain fatty acids; MAPK, mitogen-activated protein kinase; JAK/STAT, Janus kinase/signal transducer and activator of transcription; IL, interleukin; TNF-α, tumor necrosis factor-alpha.

Overall, dietary interventions influence cytokine regulation through multiple pathways, including direct molecular interactions with immune receptors and indirect modulation via gut microbiota. These mechanisms underscore the potential of targeted dietary strategies to alleviate cytokine-driven diseases like psoriasis (Figure 2).

## 6. Clinical Implications and Future Directions

Clinical trials and cohort studies provide valuable insights into the relationship between dietary interventions and cytokine modulation in psoriasis [89]. Numerous studies have demonstrated that specific diets, such as the Mediterranean or ketogenic diet, can reduce levels of pro-inflammatory cytokines like TNF-α and IL-17, while enhancing anti-inflammatory cytokines such as IL-10 [27,52,58,62]. These findings often correlate with clinical improvements, including reduced PASI scores and better quality of life [46,47,128]. However, variability in study design, population characteristics, and dietary adherence presents challenges in drawing definitive conclusions. Despite these limitations, the accumulating evidence supports the integration of diet as an adjunctive therapy in psoriasis management.

Translating cytokine modulation through diet into clinical practice faces several challenges. Individual responses to dietary changes can vary due to genetic, environmental, and lifestyle factors, making it difficult to establish one-size-fits-all guidelines [89]. Additionally, ensuring patient adherence to specific diets over the long term can be complex, as dietary changes require sustained motivation and behavioral shifts [129,130]. Integrating dietary interventions into standard psoriasis care is also complex, given that pharmacological treatments remain the primary approach [131]. To effectively counsel patients on dietary strategies, healthcare providers require adequate training and resources. Multidisciplinary collaboration among dietitians, dermatologists, and primary care physicians may be essential [1,10].

Identifying reliable biomarkers to monitor the effectiveness of dietary interventions in psoriasis is an emerging research area. Biomarkers such as serum cytokine levels (e.g., TNF-α, IL-17, IL-23), gut microbiota composition, and metabolic markers (e.g., SCFAs, ketone bodies) could provide insights into the impact of diet on immune function and inflammation [1,10]. Non-invasive markers, such as those derived from blood or stool samples, are particularly valuable for routine monitoring [132]. Developing biomarker panels tailored to dietary interventions could enable clinicians to personalize treatments, monitor progress, and optimize therapeutic outcomes [133,134].

Future research in psoriasis management should focus on personalized nutrition and precision medicine approaches. Advances in genomics, microbiomics, and metabolomics offer the potential to identify individual patient profiles that predict responses to specific dietary interventions [74,77,135]. This approach would allow for dietary strategies customized to genetic predispositions, cytokine profiles, and gut microbiota composition. Furthermore, combining dietary interventions with pharmacological treatments could enhance therapeutic outcomes and reduce dependence on long-term medication [136]. As the field progresses, interdisciplinary collaboration and robust clinical trials will be critical to refining these approaches and ensuring their feasibility in real-world clinical settings.

## 7. Conclusions

Dietary interventions have emerged as a promising adjunctive approach for managing cytokine dysregulation in psoriasis. Key findings highlight the role of diets such as the Mediterranean, ketogenic, and fasting-mimicking diets in modulating pro-inflammatory cytokines like TNF-α, IL-17, and IL-23 while enhancing anti-inflammatory cytokines such as IL-10. These dietary patterns not only reduce systemic inflammation but also improve clinical outcomes, including skin lesion severity and patient quality of life [75]. The mechanisms underlying these effects involve nutrient-specific interactions with immune pathways and the modulation of gut microbiota, which play a central role in regulating cytokine production. Evidence from clinical trials and cohort studies underscores the potential of dietary strategies to complement conventional therapies, offering a holistic approach to psoriasis management.

Furthermore, dietary components such as omega-3 fatty acids, polyphenols, and SCFAs derived from fiber fermentation have demonstrated their ability to attenuate inflammatory signaling pathways [97,99]. These nutrients act at the molecular level, suppressing pathways like NF-κB and enhancing regulatory mechanisms that counteract inflammation. The findings emphasize that dietary choices not only influence cytokine activity but also address comorbidities such as obesity and metabolic syndrome, which exacerbate psoriasis severity. The cumulative evidence supports the integration of dietary recommendations into psoriasis care plans, laying the foundation for individualized and sustainable therapeutic strategies.

The implications for future research and clinical applications are significant. Studies should focus on elucidating the precise molecular pathways influenced by specific diets and identifying biomarkers that can track the efficacy of dietary interventions. These biomarkers, including serum cytokine levels and gut microbiota profiles, will enable more targeted and effective dietary strategies tailored to individual patient needs. Research should also address the long-term sustainability and safety of these dietary approaches, to ensure their seamless integration into standard care. Additionally, understanding how dietary interventions interact with existing pharmacological treatments could unlock synergistic benefits, optimizing overall patient outcomes.

Clinical applications of these findings demand a shift toward personalized nutrition and precision medicine. Advances in genomics and metabolomics could help identify patient subgroups most likely to benefit from specific dietary changes. This personalized approach could improve adherence, as patients experience more tangible benefits tailored to their unique biological profiles. Integrating dietary counseling into routine care, supported by interdisciplinary teams and accessible resources, will be essential for success. As research continues to expand, the incorporation of dietary strategies into psoriasis treatment protocols could transform care by targeting both symptoms and the underlying inflammatory mechanisms driving the disease.
